# A Sparse Dictionary Learning-Based Adaptive Patch Inpainting Method for Thick Clouds Removal from High-Spatial Resolution Remote Sensing Imagery

**DOI:** 10.3390/s17092130

**Published:** 2017-09-15

**Authors:** Fan Meng, Xiaomei Yang, Chenghu Zhou, Zhi Li

**Affiliations:** 1 State Key Laboratory of Resources and Environmental Information System, Institute of Geographic Sciences and Natural Resources Research, Chinese Academy of Sciences, Beijing 100101, China; mengf@lreis.ac.cn (F.M.); zhouch@lreis.ac.cn (C.Z.); 2 Jiangsu Center for Collaborative Innovation in Geographical Information Resource Development and Application, Nanjing 210023, China; 3 State Key Laboratory of Desert and Oasis Ecology, Xinjiang Institute of Ecology and Geography, Chinese Academy of Sciences, Urumqi 830011, China; lizhi@lreis.ac.cn; 4 University of Chinese Academy of Sciences, Beijing 100049, China

**Keywords:** sparse representation, dictionary learning, image inpainting, thick clouds removal, high resolution remote sensing image

## Abstract

Cloud cover is inevitable in optical remote sensing (RS) imagery on account of the influence of observation conditions, which limits the availability of RS data. Therefore, it is of great significance to be able to reconstruct the cloud-contaminated ground information. This paper presents a sparse dictionary learning-based image inpainting method for adaptively recovering the missing information corrupted by thick clouds patch-by-patch. A feature dictionary was learned from exemplars in the cloud-free regions, which was later utilized to infer the missing patches via sparse representation. To maintain the coherence of structures, structure sparsity was brought in to encourage first filling-in of missing patches on image structures. The optimization model of patch inpainting was formulated under the adaptive neighborhood-consistency constraint, which was solved by a modified orthogonal matching pursuit (OMP) algorithm. In light of these ideas, the thick-cloud removal scheme was designed and applied to images with simulated and true clouds. Comparisons and experiments show that our method can not only keep structures and textures consistent with the surrounding ground information, but also yield rare smoothing effect and block effect, which is more suitable for the removal of clouds from high-spatial resolution RS imagery with salient structures and abundant textured features.

## 1. Introduction

During the past decades, remote sensing (RS) images have been commonly adopted in many applications, like scene interpretation, land-use classification, land-cover change monitoring, and atmospheric environment surveying. Especially with the high demand for finer earth observation, high-spatial resolution RS imagery plays an increasingly important role in landscape information interpretation, which provides precise and abundant representation of surface features (e.g., geometrical structures and textured patterns). However, owing to the influence of observation conditions, optical imageries from satellite sensors are often corrupted by clouds, which limits the availability of RS data. Hence, removing clouds to recover real ground information is of great significance for practical application purposes.

Many studies have been dedicated to coping with the problem of cloud removal, to reduce or eliminate the influence caused by clouds. To some extent, it is equivalent to the image inpainting problem [1] as long as clouds are accurately detected (see [2,3,4,5] for more details on cloud detection). Removing clouds is essentially a process of recovering the missing information, and the existing methods can fall into three classes [6,7,8]: one class is multispectral complementation based; the second is multitemporal complementation based; and spatial-complementation based methods. Shen et al. [9] and Gladkova et al. [10] followed the way of multispectral complementation to recover the missing MODIS data by utilizing spectral correlation between the corrupted band and other cloud-free bands. Li et al. [11] adopted shortwave infrared images to reconstruct the visible image via the fusion scheme based on variational gradients, which is only suitable for haze and thin clouds removal but cannot work for thick clouds that always contaminate whole bands in the imageries. Moreover, Shen et al. [12] presented a compressed sensing (CS) based inpainting approach that adaptively weights the clear bands in terms of spectral importance to restore Aqua MODIS band 6. Nevertheless, all these methods are generally confined to the spectral compatibility [6,7] and tend to have trouble removing thick cloud.

By contrast, it seems more attractive to take multitemporal complementation into consideration to recover the missing information sheltered by clouds. In this way multitemporal RS images acquired at different times and over the same area are employed. Tseng et al. [13] developed the multi-scale wavelet based fusion approach to recover the cloud zones in the base image using multitemporal cloud-free images. In references [7,14] somewhat similar ideas were used to generate cloud-free images. Regression analysis [15] was also introduced to reconstruct the missing regions aided by just one reference image from another period. The neighborhood similar pixel interpolator (NSPI) method originally proposed to repair gaps in Landsat7 ETM+ images was then modified by Zhu et al. [16] for thick clouds removal. Recently, sparse representation was incorporated into the multitemporal complementation based approaches, producing quite promising results [8,17,18,19,20]. As pointed out in references [8,14,21], these approaches cannot work well when suffering from severe overlapping of cloud cover, significant spectral differences caused by atmospheric conditions, and rapid changes of land use. In addition, most of these methods cannot well preserve the spatial continuity of the ground features which will be abundantly and finely revealed in high-spatial resolution RS images.

The cloud removal approaches of spatial-complementation category are mainly developed based on the image inpainting technique, which utilize the known ground information in the cloud-free regions to infer the cloudy parts. The goal of image inpainting is to seamlessly reconstruct a visually pleasant and consistent image [21]. It should be mentioned that total variation (TV) [22] and partial differential equations (PDE) [23] were both introduced to the inpainting problem, which are sorted as the diffusion-based approaches and achieve excellent results when filling in smaller missing regions without textures. To repair larger missing regions and preserve texture features, exemplar-based inpainting algorithms [24,25] deriving from the texture synthesis technique were employed to inpaint images at the patch level. Xu et al. [26] presented a patch-sparsity based inpainting approach which can produce sharp structures and consistent textures to surrounding information. Sparse representation also gives out a competitive solution to this problem in the way of spatial complementation [27,28,29,30,31]. Bandelet transform [21], maximum a posteriori (MAP) [32], patch filling [33], and nonlocal TV [34] were all adopted to inpaint RS images. Furthermore, Markov random field (MRF) [35] and newly developed low-rank tensor completion (LRTC) [36,37] can also be applied to cloud removal research. Resorting to the known information of the image, image inpainting techniques can produce visually pleasant results that are suitable for cloud-free visualization [6,7]. However, when repairing larger missing regions with composite structures and textures, most existing methods will discard some detailed features of the ground information, leading to smoothing effects and block effects.

As stated above, sparse representation has been gradually deployed into the image restoration field and proven to be appropriate for recovering large-area missing information recently [38]. Inspired by this idea and the latest progress on exemplar-based image inpainting, we present a dictionary-learning based adaptive inpainting approach via patch propagation. Due to the widespread sparsity of RS images, the feature dictionary was learned from exemplars in the cloud-free regions to infer the cloud-contaminated parts by sparse representation afterwards. In the patch selection stage, structure-sparsity based patch priority was employed to encourage the repairing of corrupted patches located at image structures, which can keep the continuity of structures. As for the patch inpainting stage, a neighborhood-consistency constraint with adaptive parameters was considered to construct the $l_{0}$-norm minimization model for retrieving the missing information beneath thick clouds, which would guarantee the consistency of synthesized textures with the surrounding information. To solve the optimization model and reconstruct complete information from incomplete measurements, a modified orthogonal matching pursuit (OMP) algorithm was put forward in this paper. Through simulated and real experiments on thick clouds removal from high-spatial resolution RS images, the proposed method exhibits a superior performance over that of some existing mainstream approaches, which can well preserve the continuity of filled structures and the consistency of synthesized textures, yielding rare smoothing effect and edge effect.

The rest of this paper is organized as follows. We start by introducing some preliminary knowledge on exemplar-based inpainting and sparse dictionary learning in Section 2. The optimization model for adaptive patch inpainting and our modified OMP algorithm are described in Section 3, where the thick-cloud removal scheme for RS imagery is designed. In Section 4, we demonstrate the experiments and compare with some existing inpainting approaches. Finally, we conclude this paper in Section 5.

## 2. Preliminaries

### 2.1. Exemplar-Based Image Inpainting

Generally, an image is composed of structures and textured features, where structures constitute the main sketches in the image (e.g., contours and edges) and textures are image regions with similar feature statistics or homogenous patterns (including smooth areas) [26]. The main thought of exemplar-based image inpainting is to inwardly propagate image information from the source regions (i.e., known parts) into the target regions (i.e., missing parts) at the patch level. For each iteration of patch propagation, patch selection and patch inpainting are two primal operations. In patch selection, the patch on the target region boundary with the highest priority is chosen for further inpainting. Patch priority is designed to urge completion of patches located at structures so that structures can be first recovered compared with textured patterns. In the patch inpainting procedure, the chosen patch is synthesized via linear combination of candidate exemplars from the source region [24,25,26].

However, most existing algorithms of patch inpainting search the candidate exemplars globally to infer the missing patches in target regions, leading to too high computational cost and poor representation ability. So, we introduce the K-SVD algorithm [39] to learn feature dictionary aiming to extend patch diversity, which will be described in following subsection. A good definition of patch priority should be able to better distinguish the structures and textures. Among those prior works, we focus on the structure-sparsity based priority [26]. Patch structure sparsity is defined by the sparsity of a patch’s nonzero similarities to its neighboring patches, which can better measure whether a patch is on structures or not.

Given an image I with source region Ω and target region $\bar{\Omega }$, the goal of image inpainting is to repair the region $\bar{\Omega }$ utilizing known information in region Ω. We use $\partial \bar{\Omega }$ to denote the boundary of region $\bar{\Omega }$ which is also called fill-front in exemplar-based inpainting approaches. $\Psi_{p}$ denotes the patch centered at pixel p, and N(p) means the neighboring window which is also centered at pixel p and with larger size than patch $\Psi_{p}$.

Suppose $\Psi_{p}$ is a patch on fill-front $\partial \bar{\Omega }$, its neighboring patches $\Psi_{p_{j}}$ are defined as patches which are located in region Ω and with center $p_{j}$ in the neighboring window N(p), i.e., $p_{j}$ belongs to the set

(1) $$N_{s}(p)=\{p_{j}:p_{j}\in N(p)\;and\;\Psi_{p_{j}}\subset \Omega \}.$$

As to patch $\Psi_{p}$, its structure sparsity is defined by

(2) $$S(p)=\sqrt{\left(\sum_{p_{j}\in N_{s}(p)}w_{p,p_{j}}^{2}\right)\cdot \frac{\left|N_{s}(p)\right|}{\left|N(p)\right|}},$$

where $w_{p,p_{j}}$ denotes the normalized similarity between $\Psi_{p}$ and $\Psi_{p_{j}}$, and |⋅| is the number of elements in a set. Structure sparsity S(p) reaches the maximal value, while the patch similarities are distributed in the sparsest form.

### 2.2. Sparse Dictionary Learning

Sparse representation has become an increasingly attractive research hotspot in recent years [39,40,41,42]. For the over-complete dictionary $D\in R^{n\times K}$, whose columns are prototype signal-atoms $d_{j}\in R^{n}(j=1,2,\cdots ,K)$, the target signal $y\in R^{n}$ can be represented as a sparse linear combination of these atoms. To be more specific, y can be approximated as y≈Dx which satisfies ${\left\|y-Dx\right\|}_{p}\leq \varepsilon$, where the vector $x\in R^{K}$ contains the representation coefficients of signal y. We set p=2 in the paper.

Suppose n<K and D is full rank, there exist innumerable solutions to this representation problem. So, a sparsity constraint is imposed on the problem, and then we can obtain the solution by

(3) $$\underset{x}{\min}{\left\|y-Dx\right\|}_{2}^{2}\;\;s.t.{\left\|x\right\|}_{0}\leq T,$$

where ${\left\|\cdot \right\|}_{0}$ denotes the $l_{0}$-norm and T limits the sparsity of representation coefficients. As we know, computing the optimal solution to (3) is the nondeterministic polynomial-time hard (NP-hard) problem. Therefore, some algorithms that approximately solve this problem were put forward. Matching pursuit (MP) and orthogonal MP algorithms [43] are the simplest ones. Moreover, basis pursuit is also a representative algorithm for solving the problem by replacing the $l_{0}$-norm with $l_{1}$-norm [44]. The focal underdetermined system solver (FOCUSS) adopts $l_{p}$-norm with p≤1 as a replacement for the $l_{0}$-norm [45].

Considering a set of signals ${\left\{y_{i}\right\}}_{i=1}^{N}(N\gg K)$, there exists a dictionary D providing the sparse solution $x_{i}$ for each signal $y_{i}$. The dictionary learning problem is to find the optimal dictionary by solving

(4) $$\underset{D,X}{\min}\{{\left\|Y-DX\right\|}_{F}^{2}\}\;s.t.\forall i,{\left\|x_{i}\right\|}_{0}\leq T_{0},$$

where $Y=\left[y_{1},y_{2},\cdots ,y_{N}\right]$, $X=\left[x_{1},x_{2},\cdots ,x_{N}\right]$ and ${\left\|\cdot \right\|}_{F}$ means the Frobenius norm. The dictionary learning algorithm based on K-SVD [39] is the generalization of K-means clustering algorithm, which iteratively alternates between sparse representation of signal examples and the updating of dictionary atoms one by one.

## 3. Methodology

### 3.1. Adaptive Patch Inpainting Model and Algorithm

**Figure 1:**
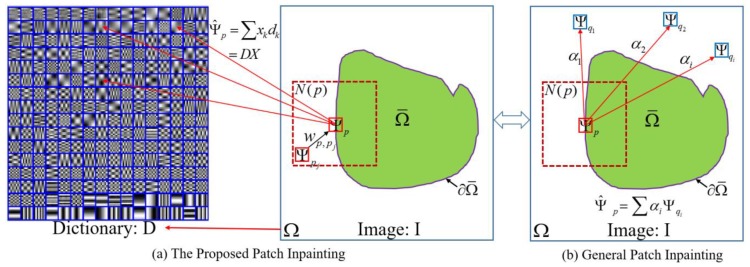
The Illustration of Sparse Dictionary Learning Based Patch Inpainting.

The exemplar-based inpainting approach generally synthesizes a missing patch by linear combination of several of the top most similar exemplars from the source regions. Unfortunately, these similar exemplars cannot perfectly describe the distinctions from the patch to be inpainted, which will deteriorate the generalization ability of linear combination. Besides, existing patch inpainting algorithms cannot yield adaptive inpainting results for each missing patch according to the neighboring characteristics, leading to inconsistency with the surrounding structures and textures in appearance. The illustration of our adaptive patch inpainting approach based on sparse dictionary learning is shown in Figure 1a, where feature dictionary D is learned from exemplars in the source region Ω to estimate the missing patch $\Psi_{p}$ via sparse representation, and neighboring patches ${\left\{\Psi_{p_{j}}\right\}}_{p_{j}\in N_{s}(p)}$ will be used to constrain the appearance of ${\hat{\Psi }}_{p}$ (i.e., the estimated patch of $\Psi_{p}$) to maintain a consistent neighborhood. Figure 1b presents the general patch inpainting technique, where several candidate exemplars are utilized to infer the estimated patch.

#### 3.1.1. Patch Priority

The filling-in order of missing patches on the fill-front is of great importance to the inpainting result. To preserve the coherence of structures, patch priority is designed to firstly select patches on image structures for further inpainting, since textured features are prone to be synthesized by exemplars or dictionary in the framework of sparse representation. Due to better discrimination of whether or not a patch is on structures, structure sparsity is hereby employed to determine the patch priority. In our paper, the similarity between patch $\Psi_{p}$ and its neighboring patch $\Psi_{p_{j}}(p_{j}\in N_{s}(p))$ is calculated by

(5) $$w_{p,p_{j}}=\frac{1}{Z(p)}\exp(-\frac{d(\pi_{\Omega }(\Psi_{p}),\pi_{\Omega }(\Psi_{p_{j}}))}{\sigma^{2}}),$$

where d(⋅,⋅) refers to the mean squared distance and Z(p) denotes the normalization constant so that $\sum_{p_{j}\in N_{s}(p)}w_{p,p_{j}}=1$. In (5), the linear operator $\pi_{\Omega }:R^{m\times n}\to R^{m\times n}$ keeps the elements in the index set Ω unchanged and sets those outside Ω zeros, and the set Ω corresponds to the indices of the known elements in patch $\Psi_{p}$. Similarly, as for the operator $\pi_{\bar{\Omega }}$, the index set $\bar{\Omega }$ is determined by the missing elements in $\Psi_{p}$. Under the guarantee of S(p), the patches on image structures will hold higher priority for further inpainting compared to the ones in textured parts.

Finally, the patch priority can be calculated by

(6) $$P(p)=T_{\left[\zeta ,1\right]}(S(p))\cdot C(p).$$

$C(p)=\sum_{q\in \Psi_{p}\cap \Omega }c(q)/|\Psi_{p}|$ denotes the confidence of $\Psi_{p}$, which means the reliability of intensity in the patch [24]. Herein, c(q) is the confidence of pixel q, which is initialized as 1 in the source region and 0 in the target region. After each iteration of patch propagation, the confidence of the newly inpainted pixels in the selected patch will be updated as patch confidence C(p). $T_{\left[\zeta ,1\right]}$ is a linear transform of S(p) from the initial interval $\left[\sqrt{1/\left|N(p)\right|},\sqrt{\left|N_{s}(p)\right|/\left|N(p)\right|}\right]$ to [ζ,1], which is necessary to make S(p) vary at a comparable scale with C(p). As a result, patch priority will encourage the repair of the patches on image structures and with larger confidence first.

#### 3.1.2. The Optimization Model for Patch Inpainting

In this subsection, the optimization model of adaptive patch inpainting under the neighborhood-consistency constraint will be put forward, which can yield continuously sharp structures and clearly fine textures that are consistent with the surrounding information. To extend the diversity of the patch $\Psi_{p}$, feature dictionary D is constructed by the K-SVD algorithm to sparsely represent the missing patch. Feature dictionary is four times redundant in our implementation so that the missing patches can be better inferred. The size of atoms in the dictionary is generally set to 8×8 pixels or 16×16 pixels in the paper, which will be specifically discussed in Section 4.

Given the patch $\Psi_{p}$ to be inpainted and dictionary D, $\Psi_{p}$ is approximated as a sparse linear combination of D, i.e.,

(7) $${\hat{\Psi }}_{p}=\sum x_{k}d_{k}=DX.$$

Then, the missing part of patch $\Psi_{p}$ can be completed by the corresponding part in ${\hat{\Psi }}_{p}$, i.e., $\pi_{\bar{\Omega }}(\Psi_{p})=\pi_{\bar{\Omega }}({\hat{\Psi }}_{p})$. To obtain the sparsest solution X, the problem can come down to $l_{0}$-norm minimization problem (i.e., $\min{\left\|X\right\|}_{0}$) by constraining the appearance of the estimated patch ${\hat{\Psi }}_{p}$.

Constraints on the known part Ω and missing part $\bar{\Omega }$ will be taken into consideration. Firstly, the estimated patch ${\hat{\Psi }}_{p}$ should approximate the missing patch $\Psi_{p}$ over the known elements, i.e.,

(8) $${\left\|\pi_{\Omega }({\hat{\Psi }}_{p})-\pi_{\Omega }(\Psi_{p})\right\|}_{F}^{2}\leq \varepsilon ,$$

where ε denotes the error tolerance of this sparse representation. Furthermore, the newly filled elements in patch ${\hat{\Psi }}_{p}$ should coincide with the neighboring image features, so the neighborhood-consistency constraint is considered to further restrict the appearance of ${\hat{\Psi }}_{p}$. Herein, the neighboring patches $\Psi_{p_{j}}(p_{j}\in N_{s}(p))$ with the corresponding similarities $w_{p,p_{j}}$ are employed again to constrain the sparse representation over the missing elements, i.e.,

(9) $${\left\|\pi_{\bar{\Omega }}({\hat{\Psi }}_{p})-\pi_{\bar{\Omega }}(\sum_{p_{j}\in N_{s}(p)}w_{p,p_{j}}\Psi_{p_{j}})\right\|}_{F}^{2}\leq \eta ,$$

where η≥ε is the error tolerance for the missing part. Suppose η=ε/β(0<β≤1) where β is the balance factor that balances the strength of constraints between (8) and (9), the two types of constraints can be reformulated as

(10) $$\left\{ \begin{array}{l} {\left\|\pi_{\Omega }(D)X-\pi_{\Omega }(\Psi_{p})\right\|}_{F}^{2}\leq \varepsilon \\ {\left\|\pi_{\bar{\Omega }}(\sqrt{\beta }D)X-\pi_{\bar{\Omega }}(\sqrt{\beta }\sum_{p_{j}\in N_{s}(p)}w_{p,p_{j}}\Psi_{p_{j}})\right\|}_{F}^{2}\leq \varepsilon . \end{array}\right.$$

In (10), we use $\pi_{\Omega }(D)$ to denote the $\pi_{\Omega }$ operation on each atom of D, i.e., $\pi_{\Omega }(D)=(\pi_{\Omega }(d_{1}),\cdots ,\pi_{\Omega }(d_{k}),\cdots )$, and so is the case with $\pi_{\bar{\Omega }}(\sqrt{\beta }D)$.

As we know, in each patch inpainting stage, the selected patch is within a different image region, so the balance factor should be adaptively adjusted according to the neighboring characteristics. In general, when patch $\Psi_{p}$ is located at structures, β should be turned down; if $\Psi_{p}$ is in a textured region, the balance factor will increase. We design an adaptive scheme to adjust the balance factor in view of patch structure sparsity, i.e.,

(11) $$\beta =\frac{1}{C\cdot T_{\left[\zeta ,1\right]}(S(p))},$$

where ζ=0.2 and C=6 to satisfy 0<β≤1 in the paper. The constraints in (10) can be formulated in a more compact form

(12) $${\left\|D_{new}X-Y\right\|}_{F}^{2}\leq \varepsilon ,$$

where $D_{new}=\pi_{\Omega }(D)+\pi_{\bar{\Omega }}(\sqrt{\beta }D)$ and $Y=\pi_{\Omega }(\Psi_{p})+\pi_{\bar{\Omega }}(\sqrt{\beta }\sum_{p_{j}\in N_{s}(p)}w_{p,p_{j}}\Psi_{p_{j}})$. Finally, the sparsest solution to X will be obtained by solving this constrained optimization model

(13) $$\min{\left\|X\right\|}_{0},\;s.t.{\left\|Y-D_{new}X\right\|}_{F}^{2}\leq \varepsilon .$$

It is equivalent to the model of (3), which can be solved via the modified versions of algorithms available for (3).

#### 3.1.3. Modified OMP and the Inpainting Algorithm

To solve the optimization model of (13), we present a modified version of OMP algorithm owing to its practicability and simplicity. Generally, the basic idea is to normalize the newly computed dictionary $D_{new}$, which can better guide the selection of optimal matching atoms to approximate the newly produced patch Y; and then, the representation coefficients $X_{new}$ for patch Y under the normalized dictionary $D_{norm}$ can be obtained by the OMP algorithm; finally, the solution to X will be retrieved from $X_{new}$, the index set of selected atoms and the corresponding norms of atoms in $D_{new}$. As a result, the unknown pixels of patch $\Psi_{p}$ can be completed by corresponding pixels in the reconstructed patch ${\hat{\Psi }}_{p}$ from (7).

**Figure 2:**
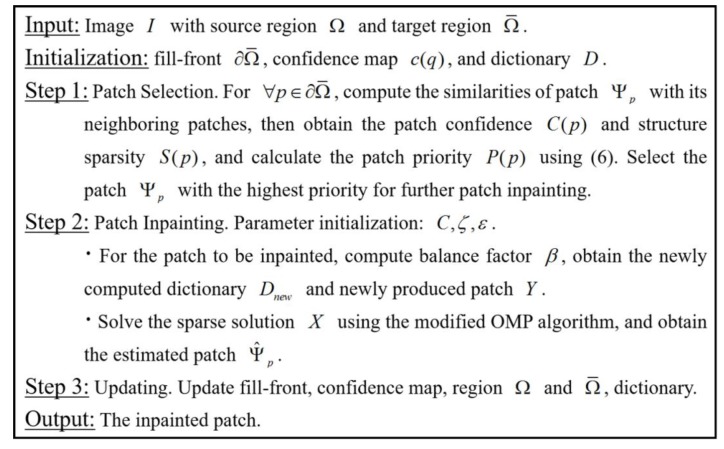
The Procedure of Adaptive Patch Inpainting Algorithm.

In summary, the overall algorithm for adaptive patch inpainting is listed in Figure 2, where the procedures of patch selection and inpainting will be mainly involved. In our implementation, the size of the neighboring window N(p) is generally set as five times the size of patch $\Psi_{p}$, which can achieve a nice tradeoff between the computational cost and neighborhood consistency; the balance factor β in (10) may be affected by the ratio between the number of unknown pixels in $\Psi_{p}$ and that of known pixels (denoted by r), so $\beta^{\prime }=\beta /r$ will be considered as a substitute for β.

### 3.2. Thick Clouds Removal Scheme for RS Imagery

When thick clouds appear in an area, the ground information may be completely contaminated, and bright-white features in clustering pattern will be revealed in optical RS image due to the high reflectance intensity of thick clouds. Luckily, RS imagery is prone to obtain a wide range of surface information, so there will always be lots of similar information within an image because of the spatial autocorrelation. This similar information includes not only the local neighborhood similarity but also the nonlocal similarity [34], which makes it possible to reconstruct the missing information utilizing the ground information in cloud-free (i.e., source) region. In light of this, the adaptive patch inpainting method based on sparse representation is applied to the problem of thick clouds removal, especially for high-spatial resolution RS image that contains salient geometric structures and abundant textured features.

Above all, the detection of thick clouds is of great significance to subsequent clouds removal, which is beyond the scope of the main topic in our paper. In short, we employ the threshold method and region growth to extract missing region, on account of high reflectance intensity and clustering characteristics of thick clouds. Once the target region $\bar{\Omega }$ is obtained, the fill-front $\partial \bar{\Omega }$ of the cloudy region is then extracted by the mathematical morphology method. Specifically, we first adopt morphological close operator with square structure element of the size 3×3 pixels to fill in small holes in the target region, aiming to avoid the tracing of unwanted edge points and too high computational cost in patch selection stage; and then, edge points of the preprocessed target region can be tracked by the operation $imclose(\bar{\Omega },se)-imerode(imclose(\bar{\Omega },se),se)$, where the functions imclose and imerode denote morphological close and erosion operator respectively, and se means the structure element.

**Figure 3:**
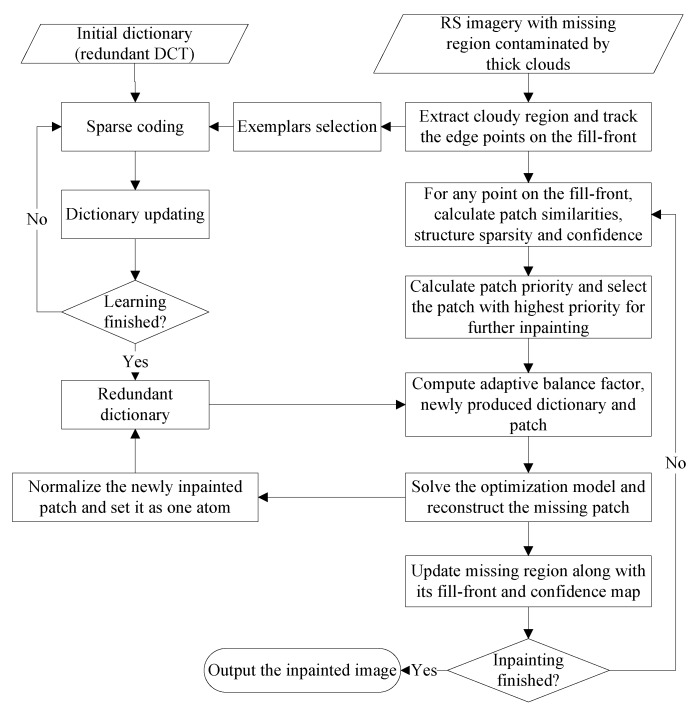
The Flowchart of Our Thick Clouds Removal Scheme.

Afterwards, the feature dictionary is learned from randomly selected exemplars in the cloud-free region Ω. It should be noted that in our paper, feature dictionary D will be augmented by the newly filled patch after each iteration of patch inpainting, which may expand the diversity of dictionary to better inpaint the remaining patches. In the following procedure, an iterative operation of adaptive patch inpainting will be performed. That is to say, repeat the patch selection and patch inpainting until the missing region contaminated by thick clouds is completely repaired. After each iteration, the target region $\bar{\Omega }$ along with its fill-front $\partial \bar{\Omega }$, the confidence map c(q) and the missing pixels in patch $\Psi_{p}$ should be updated accordingly. The whole workflow of thick clouds removal based on adaptive patch inpainting algorithm is presented in Figure 3.

## 4. Experiments and Discussion

This section is devoted to the experimental analysis and discussion of our adaptive patch inpainting scheme for removing clouds from high resolution RS images. Some existing methods (e.g., texture synthesis technique, morphological component analysis (MCA) [27,46], MRF [35]) and most related works [24,26] will be employed to restore the missing information corrupted by manually appended masks and real clouds. Additionally, the recently developed LRTC [36,37] will also be applied to the comparison experiments. MATLAB implementation of the MCA algorithm that is available as a part of the MCALab package can be downloaded at http://www.greyc.ensicaen.fr/~jfadili/demos/WaveRestore/downloads/mcalab/Home.html, and the code for the MRF method is available at http://www.gris.informatik.tu-darmstadt.de/research/visinf/software/index.en.htm.

Comparison results are shown in the form of statistical index tables and visual effects. To evaluate the quality of the restoration results, peak signal to noise ratio (PSNR) and structural similarity (SSIM) are adopted for objective evaluation and subjective visual evaluation, respectively. Given an image $I\in {\left[0,255\right]}^{m\times n}$, the PSNR index of the restored image $\hat{I}$ is defined as follows:

(14) $$PSNR(I,\hat{I})=10\log_{10}\frac{255^{2}}{MSE},MSE={\left\|I-\hat{I}\right\|}_{F}^{2}/(m\times n).$$

The SSIM index is the metric that better corresponds to the subjective quality of visual perception, which can be formulated as

(15) $$SSIM(I,\hat{I})=\frac{\left(2\mu_{I}\mu_{\hat{I}}+C_{1})(2\sigma_{I\hat{I}}+C_{2}\right)}{\left(\mu_{I}^{2}+\mu_{\hat{I}}^{2}+C_{1})(\sigma_{I}^{2}+\sigma_{\hat{I}}^{2}+C_{2}\right)}$$

(see [47] for more details). The dynamic range of SSIM is [0,1], which means a better recovery performance with the SSIM closer to the value 1.

**Figure 4:**
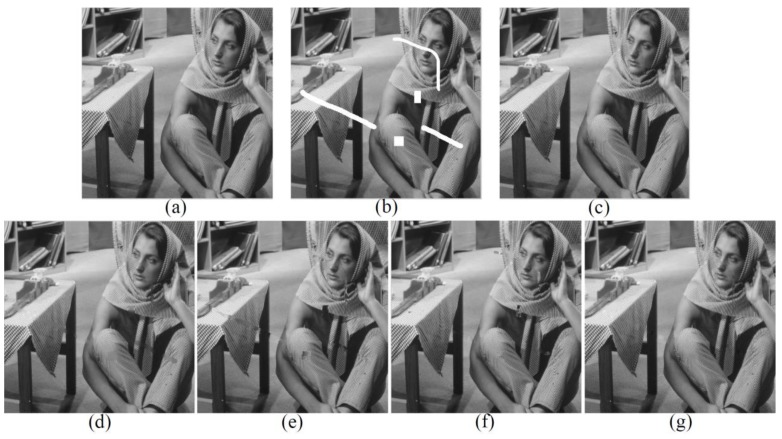
Comparison of Visual Effects on Barbara Image. (**a**) Original Image; (**b**) Corrupted Image; (**c**) Result of Ours; (**d**) Result of MRF; (**e**) Result of Texture Synthesis; (**f**) Result of [24]; (**g**) Result of MCA.

We’ll start with the inpainting experiment on the Barbara image—commonly used in the field of image processing—to validate the high performance of our adaptive patch inpainting algorithm. As shown in Figure 4, the original image of size 512×512 pixels contains abundant textures, which is then corrupted by white scratches and blocks in Figure 4b. Figure 4c–g are the inpainting results of the proposed method, MRF, texture synthesis, exemplar-based [24] and MCA, respectively. Dictionaries used in the MCA are curvelets for the cartoon layer and 2-dimensional cosine packets for the texture layer. In all experiments, parameters of each method have been tuned to yield the best results. It is clear that the proposed approach greatly outperforms other methods, which keeps better consistency with the surrounding textures and avoids smooth effect and artifacts in appearance. The repaired results of texture synthesis and exemplar-based method [24] are not visually pleasant, while in Figure 4d, smooth effect and block effect appear in the inpainted regions. By contrast, the MCA method can produce a visually reasonable result, yet somewhat smooth effects in the textured region and slight vestiges of the structures still remain. The values of PSNR and SSIM indices given in Table 1 also demonstrate that our inpainting method performs better not only according to an objective index, but also to subjective visual effect. Note that in our paper, the statistical indices are only computed for the recovered regions.

**Figure 5:**
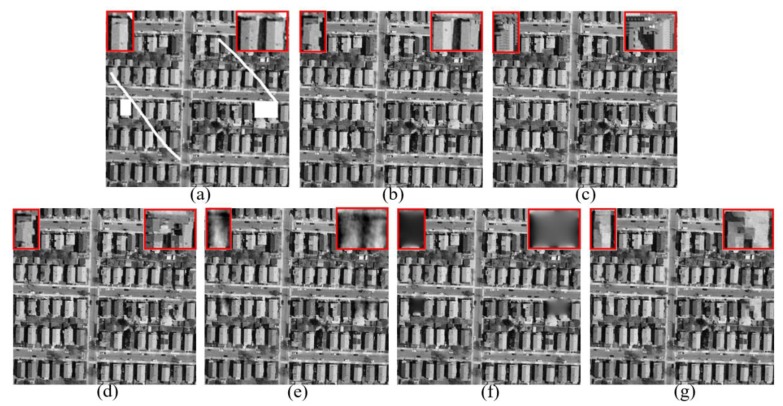
Comparison of Visual Effects on Aerial Image. (**a**) Corrupted Buildings Image; (**b**) Our Result Using 16 × 16 Patch; (**c**) Our Result Using 8 × 8 Patch; (**d**) Result of [26] Using 16 × 16 pixels Patch; (**e**) Result of MCA; (**f**) Result of MRF; (**g**) Result of Texture Synthesis.

In the following experiments, we perform our method on RS images. In Figure 5a, the red-band aerial image of buildings is contaminated by some masks, two of which are rectangle masks with the size of 43×28 pixels and that of 43×56 pixels respectively. The original information sheltered by the two white rectangles is magnified and presented in two red panes located at the top-left and top-right part of Figure 5a, respectively. For comparison, some related inpainting methods (including the patch-sparsity based approach [26]) are utilized to remove the masks and recover the missing ground information. In addition, the proposed method is performed repeatedly when the size of dictionary atom is set to different scales (see Figure 5b,c). The inpainted results for two missing rectangle blocks using each method are enlarged and shown in the corresponding subfigures. From Figure 5, we can see that MRF-based method yields the worst result along with severely over-smooth effect and block effect, and the MCA method cannot well recover the edges or contours of the buildings. It is worth noting that the texture synthesis technique can achieve relatively better result when textured patterns are regular and repeated in the image. When the size of the dictionary atom is 16×16 pixels, the proposed method can produce better recovery results with sharp structures and consistent textures, compared to the case when the size of atom is set as 8×8 pixels. Generally, when the size of the neighboring window N(p) (five times the size of dictionary atom in our paper) is at a comparable scale with the area of the missing region, our adaptive patch inpainting algorithm can achieve the optimal performance. However, even if the size of exemplars is also set to 16×16 pixels, the recovery performance of [26] is still inferior to ours. The main reasons are that several top best matching patches cannot perfectly extend the diversity of the inpainted patches, and that the balance factor cannot be adaptively adjusted according to the neighboring characteristics.

**Figure 6:**
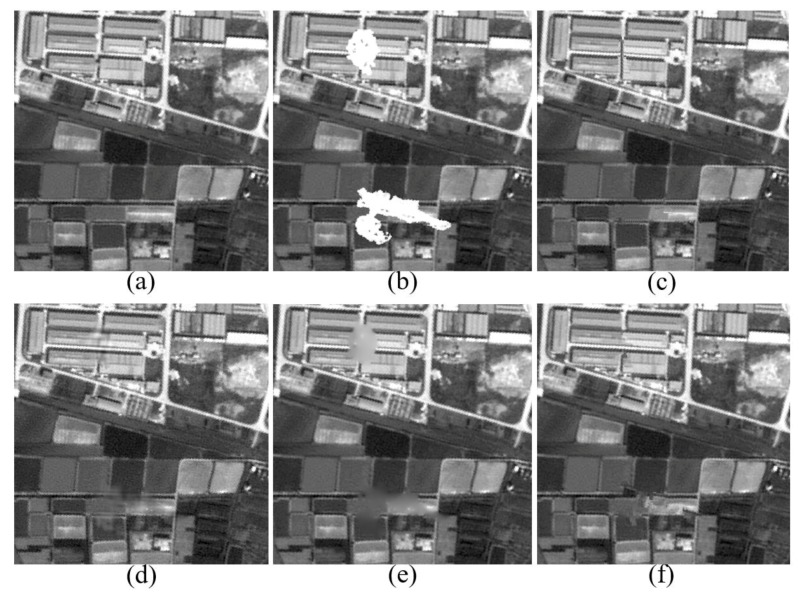
Comparison of Visual Effects on SPOT5 Panchromatic Imagery. (**a**) Original SPOT5 Image; (**b**) Corrupted by Simulated Clouds; (**c**) Result of Proposed Method; (**d**) Result of MCA; (**e**) Result of MRF; (**f**) Result of Exemplar-based.

To better demonstrate the excellent performance of our method, we do a further comparison experiment on SPOT5 panchromatic imagery with a resolution of 2.5 m. Simulated clouds are added to the original image, and the comparison of cloud removal results are shown in Figure 6. Table 2 presents the comparisons of statistical indices for the red-band aerial image and SPOT5 panchromatic imagery. Again, the results show that our method can achieve continuously sharp structures and consistently fine textures without introducing artifacts, along with higher values of PSNR and SSIM. In Figure 6c, we can observe that the contours of buildings and the ridges of farmlands are well preserved under the guarantee of structure-sparsity based patch selection and sparse-representation based patch inpainting; meanwhile, due to sparse dictionary learning and adaptive neighborhood-consistency constraint, the textured features can also be perfectly synthesized.

**Figure 7:**
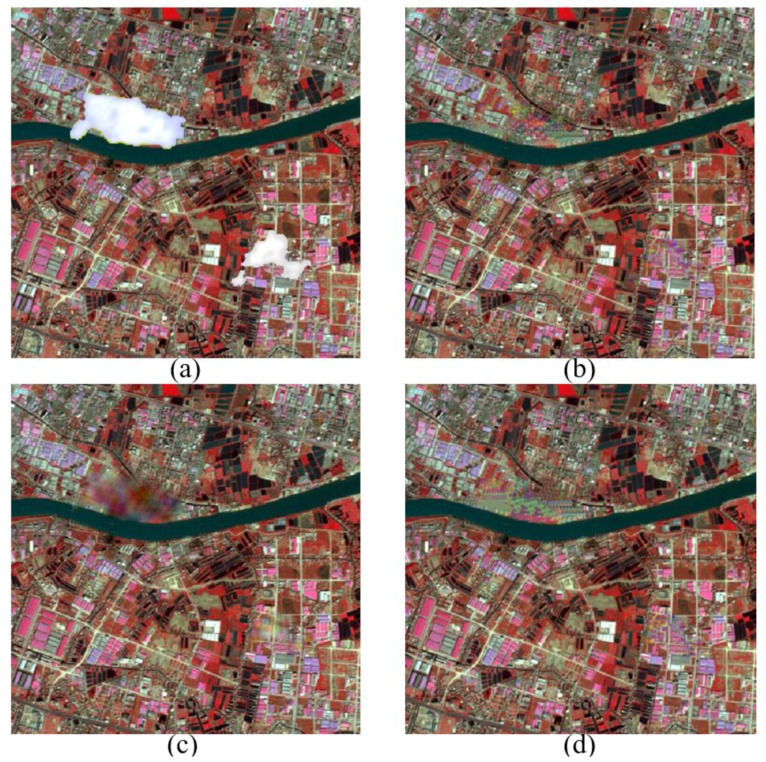
Comparison of Clouds Removal from SPOT5 Multispectral Imagery. (**a**) SPOT5 Image (displayed as false color composites) with Preprocessed Clouds; (**b**) Cloud Removal by Our Inpainting Method; (**c**) Cloud Removal by MCA [46]; (**d**) Cloud Removal by [26].

**Figure 8:**
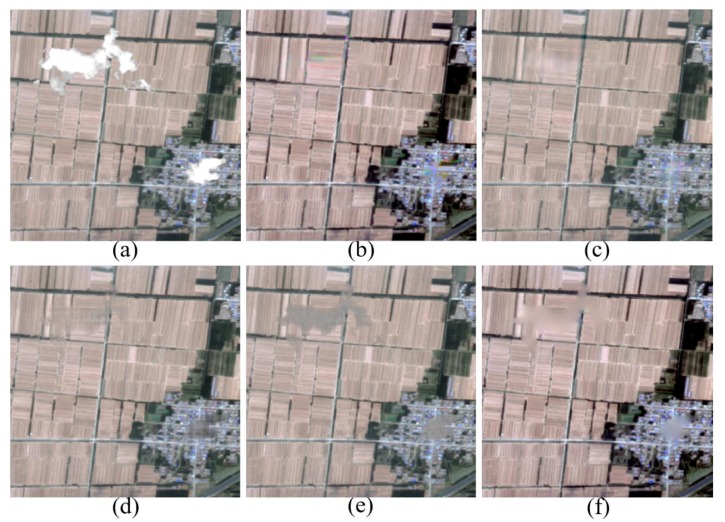
Comparison of Clouds Removal from GaoFen-2 RS Imagery. (**a**) GaoFen-2 RS Image (true color composites) with Clouds; (**b**) Cloud Removal by Our Inpainting Method; (**c**) Cloud Removal by MCA [46]; (**d**) Cloud Removal by FaLRTC [37]; (**e**) Cloud Removal by SiLRTC [37]; (**f**) Cloud Removal by MRF [35].

Furthermore, the adaptive patch inpainting method is performed on high-spatial resolution RS images contaminated by thick clouds, compared to the methods of [26], MRF [35], LRTC [37] with two solvers (i.e., SiLRTC and FaLRTC), and MCA [46]. Multispectral images are selected for the cloud removal experiments, and our method is independently run for each band of RS images. For convenience, the cloudy regions have been preprocessed by morphological operators for better extraction of missing regions and their fill-fronts. The factors of land use type and image data source are considered to validate the performance of our method. Figure 7a shows the SPOT5 image of urban area in Guangzhou, China, and Figure 7b–d are the results of clouds removal using the proposed method, MCA and [26], respectively. From Figure 7, it can be clearly seen that our method can almost perfectly recover the contours of rivers and roads, and produce consistent textures with the surrounding information. However, the approach of [26] cannot well preserve the continuity of rivers when the proportion of missing parts is large. The MCA method works worst and introduces an over-smooth effect in the textured parts. We further perform the comparison experiments of cloud removal on GaoFen-2 image of farmlands in Yucheng, Shandong province, China. Herein, GaoFen-2 refers to China’s civilian optical RS satellite with space resolutions of 1 m for panchromatic band and 4 m for multispectral bands. As shown in Figure 8, our method can yield continuous structures and consistent textures with rare smoothing effect and edge effect. By contrast, the FaLRTC algorithm can well reconstruct low-rank textures, yet it cannot work for structures.

Besides optical RS images, quantitative RS products (e.g., sea surface temperature (SST) and the normalized difference vegetation index (NDVI)) also contain missing information obscured by clouds. Moreover, since the scan line corrector (SLC) of Landsat-7 ETM+ sensor failed in 2003, wedge-shaped stripes appear in the acquired images. All these kinds of missing information greatly limit the availability of RS data. Owing to the high performance of recovering structures and textured information, our adaptive patch inpainting method can provide a promising solution to the problem of missing information reconstruction for RS data.

However, there are some limitations that need to be overcome. As we know, image features usually tend to repeat themselves both within the same scale and across different scales. Meanwhile, different features prefer different scales for the optimal representation of local information. Therefore, it is necessary to represent image features at simultaneously multiple scales. In addition, the size of patch to be inpainted should also be adaptively adjusted according to the neighboring characteristics. For example, when the patch to be inpainted is on structures, patch size should be small to finely describe detailed features and avoid smooth effect; if the patch is within textured region, the size should be large to keep integrality of spatial semantic features. As shown in Figure 8b, a little green artifact appears in the recovered farmlands, which is not semantically coherent to the surrounding textures. That is because the patch size for the inpainting stage is fixed as 8 × 8 pixels, which is suitable for the town area and most of the farmlands. The size is too small to keep the completeness of contextual information in textured regions. As a result, some missing patches in the farmlands may be synthesized by the information from small green parcels located between the farmlands and town areas. If patch size increases, smooth effect will appear in the town area. To sum up, multiscale adaptive patch inpainting schemes need to be considered.

## 5. Conclusions

In this paper, to address the problem of RS information recovery from larger missing regions contaminated by thick clouds, we propose an adaptive patch inpainting approach based on sparse dictionary learning, which can yield continuously sharp structures and consistently fine textures without introducing smoothing effect and edge effect. Our method is especially more effective for cloud removal from high-spatial resolution RS imagery that contains salient structures and abundant textured features.

Feature dictionary learning from exemplars in cloud-free regions can extend the diversity of image patches to be inpainted in the framework of sparse representation. Adaptive neighborhood-consistency constraint is introduced to formulate the novel optimization model, and modified OMP algorithm is designed to solve the problem afterwards. Experiments and comparisons are performed on RS images with simulated and real clouds, which show that our cloud removal scheme can outperform other related methods over visual effects and statistical indices. Since the research on the scale effect is of great importance to RS data, we will further investigate the algorithm of multi-scale dictionary learning from RS spatiotemporal data in the future in order to develop a multiscale adaptive patch inpainting method for missing RS information reconstruction.

## Figures and Tables

**Table 1:** Comparisons of Statistical Indices on Barbara Image.

Barbara Image	Texture Synthesis	Result of [24]	Result of MRF	Result of MCA	Result of Ours
PSNR(DB)	16.836	19.942	19.887	23.348	24.408
SSIM	0.805	0.898	0.897	0.954	0.966

**Table 2:** Comparisons of Statistical Indices on Aerial and SPOT5 Imagery.

Images	Statistical Indices	Texture Synthesis	[24]	MRF	MCA	[26]	8 × 8 pixels Patch	16 × 16 pixels Patch
Aerial	PSNR(dB)	13.663	-	10.488	13.599	13.082	13.833	15.281
	SSIM	0.632	-	0.325	0.682	0.546	0.657	0.752
SPOT5	PSNR(dB)	-	17.576	17.792	18.092	-	-	18.883
	SSIM	-	0.814	0.801	0.818	-	-	0.868
